# Research Progress on the Application of Nanoenzyme Electrochemical Sensors for Detecting Zearalenone in Food

**DOI:** 10.3390/nano15100712

**Published:** 2025-05-09

**Authors:** Guoqiang Guan, Zhiyuan Lin, Jingya Qian, Feng Wang, Liang Qu, Bin Zou

**Affiliations:** 1 School of Food and Biological Engineering, Jiangsu University, Zhenjiang 212013, China; ggqyxq@ujs.edu.cn (G.G.); 2222218077@stmail.ujs.edu.cn (Z.L.); qianjingya@ujs.edu.cn (J.Q.); fengwang@ujs.edu.cn (F.W.); 2 School of Food and Biological Engineering, Wuhu Institute of Technology, Wuhu 241003, China; 101385@whit.edu.cn

**Keywords:** zearalenone, nanoenzyme electrochemical sensors, food safety, mycotoxin detection

## Abstract

Zearalenone (ZEN) is a common mycotoxin widely found in food crops such as corn. The toxicity of ZEN is manifested as multiple hazards to reproduction, genes, cells, and immune systems. Long-term exposure may have a serious impact on health, so it has received extensive attention due to its potential harm to human and animal health. In order to ensure food safety, countries have formulated corresponding ZEN content limit standards and promoted the development of efficient and rapid detection technologies. This paper reviews the research progress of ZEN detection in food based on nanoenzyme electrochemical sensors. Firstly, the basic situation of ZEN was introduced, including its physical and chemical properties, toxicity, and related regulations and standards. Secondly, the advantages and disadvantages of traditional detection methods and new detection technologies are analyzed, and the application progress of electrochemical sensors in ZEN detection is discussed, especially aptamer electrochemical sensors, immune-electrochemical sensors, and nanoenzyme electrochemical sensors. In this paper, the advantages of nanoenzyme electrochemical sensors in ZEN detection are discussed in detail, especially in terms of sensitivity, selectivity, and rapid detection. However, nanoenzyme electrochemical sensors still face some challenges in practical applications, such as high production costs, control of signal amplification effects, and safety issues of nanomaterials. Finally, this paper looks forward to the future development direction of nanoenzyme electrochemical sensors and proposes possible solutions to further improve their stability, reduce costs, and optimize sensing performance.

## 1. Introduction

### 1.1. Overview of Zearalenone

Zearalenone (ZEN) is a mycotoxin produced by Fusarium, such as Fusarium graminearum, Fusarium graminearum, and Fusarium [1,2,3,4]. It is common in corn, wheat, and other grains and may also enter vegetable oil through contaminated vegetable oil raw materials [5,6]. ZEN is difficult to be completely removed during vegetable oil processing, so it may remain in refined oil [7,8]. Unlike other mycotoxins, ZEN has estrogen-like effects, which can interfere with the human endocrine system and lead to hormone level disorders, which may lead to a series of health problems, such as reproductive system abnormalities and endocrine-related diseases such as breast cancer. At the same time, zearalenone is also genotoxic. It can induce gene mutations and damage cells through cytotoxic effects, which may lead to cell death or dysfunction [9]. In addition, ZEN also has an inhibitory effect on the immune system, and long-term exposure may reduce the body‘s immune defense ability, thereby increasing the risk of infection and chronic diseases [10,11]. Long-term intake of contaminated vegetable oils may increase health risks [12,13], especially for groups with more vulnerable immune systems, such as children and pregnant women. Therefore, the quality of raw materials should be strictly controlled in the production of vegetable oil, and the content of ZEN in refined oil and its processing by-products should be ensured to meet food safety standards through detection.

### 1.2. Physicochemical Properties of ZEN

**Figure 1:**
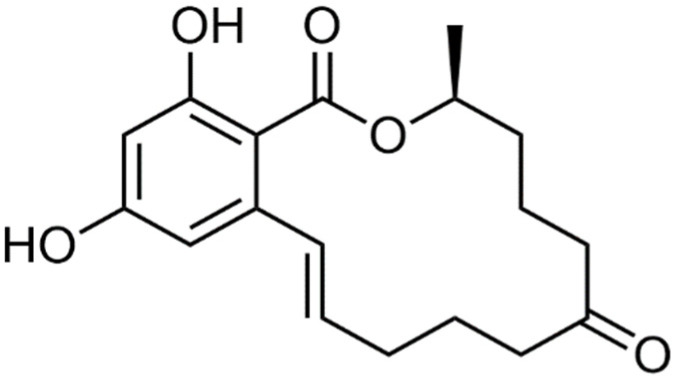
The structural formula of ZEN.

ZEN is a phenolic compound with a dihydroxybenzoate lactone structure. The chemical formula is C_18_H_22_O_5_, the structural formula is shown in Figure 1, the molecular weight is 318.364, the melting point is 164–165 °C, the boiling point is 600.4 °C, the flash point is 6 °C, the refractive index is 1.539, and the high density is 1.168 g/cm^3^ [14]. In its physical properties, ZEN has a weak polarity and is insoluble in solvents such as water and carbon tetrachloride, but it is soluble in alkaline aqueous solutions. Its solubility increases in n-hexane, benzene, acetonitrile, dichloromethane, methanol, ethanol, and acetone [15]. In its chemical properties, ZEN is easily reduced to form α-zearalenol and β-zearalenol [16].

### 1.3. Toxicity of ZEN

Because of its similar structure to estrogen, ZEN interferes with the human endocrine system, especially the estrogen receptor signaling pathway, resulting in female uterine hypertrophy, ovarian dysfunction, ovulation disorders, and abnormal embryonic development [17,18,19,20]. For men, ZEN may cause testicular atrophy, decreased sperm count and motility, and even lead to infertility. In addition, ZEN can also affect fetal development, leading to embryonic malformation or miscarriage [21,22]. Its reproductive toxicity mechanism also includes binding to estrogen receptors, interfering with hormone synthesis, etc. Long-term intake of food contaminated by ZEN poses a serious hazard to human health, especially in sensitive stages such as adolescence and pregnancy [23,24,25].

The genotoxicity of ZEN is mainly manifested by DNA damage and induction of cell mutation [26,27]. Studies have shown that ZEN can produce active metabolites during metabolism in vivo, such as α-Zearalenol and β-Zearalenol. These metabolites can bind to DNA molecules to form DNA adducts, resulting in DNA strand breaks, base mutations, or chromosome aberrations [16]. In addition, ZEN can further aggravate DNA damage by inducing oxidative stress and increasing the level of reactive oxygen species (ROS) in cells. Long-term exposure to ZEN may increase the risk of cell carcinogenesis [28,29], especially in metabolically active tissues such as the liver and kidney. Therefore, the genotoxicity of ZEN not only affects the normal function of cells but also may have a profound impact on the genetic stability of organisms.

ZEN has significant cytotoxicity and can cause cell damage through a variety of mechanisms [30]. In the case of high concentration or long-term exposure, ZEN can induce oxidative stress; generate a large number of free radicals; damage intracellular biological macromolecules, such as DNA, proteins, and lipids; and lead to cell dysfunction or death [31,32]. Specifically, ZEN can increase the level of reactive oxygen species (ROS) in cells, destroy the integrity of cell membranes, damage organelles, especially mitochondria, interfere with the energy metabolism of cells, and lead to apoptosis or necrosis [33]. In addition, ZEN may also regulate the cell cycle by activating intracellular stress signaling pathways, affecting cell division and proliferation, leading to cell proliferation inhibition or cell cycle arrest. The cytotoxicity of ZEN is not limited to a certain type of cell; it can affect many types of cells, including immune cells, liver cells, kidney cells, etc. [34], and then cause damage to various organs and systems of the body. Long-term exposure may lead to tissue damage and organ dysfunction [35].

ZEN can inhibit the immune system and affect the immune function of the body [36,37]. Studies have shown that ZEN can inhibit the function of immune cells through a variety of mechanisms, mainly affecting the activity of immune cells such as leukocytes, macrophages, and T cells [38]. ZEN can interfere with the proliferation and differentiation of immune cells and inhibit the phagocytosis of immune cells and antigen presentation function, thereby reducing the body’s immune response [39]. In addition, ZEN may also change the cytokine balance of the immune system by affecting the secretion of cytokines, leading to immune system dysfunction [40]. This immunosuppressive effect makes individuals exposed to ZEN more vulnerable to pathogens such as bacteria and viruses and may also increase the risk of autoimmune diseases.

### 1.4. Relevant Regulations and Limits of ZEN

In order to protect public health, countries and relevant institutions have set different ZEN limit standards according to their food safety regulations and epidemiological research results. China’s current limit standards for ZEN include the following: the ‘National food safety standard limit for mycotoxins in food’ (GB 2761-2017) issued in March 2017 [41], which stipulates that the maximum content of ZEN in cereals and their products is 60 μg/kg; the ‘Feed Hygiene Standard’ (GB 13078-2017) issued in October of the same year stipulates that the upper limit of ZEN content in feed raw materials and feed products is 100 μg/kg to 1500 μg/kg [42]. The European Commission Regulation (EU) NO 1881/2006 issued in 2006 sets the limit of ZEN in infant food at 20 μg/kg [43]. The Food and Agriculture Organization of the United Nations/World Health Organization Joint Committee of Experts on Food Additives stipulates that the maximum intake of ZEN per person per day does not exceed 0.5 μg/kg [44].

## 2. Overview of ZEN Detection Methods

**Figure 2:**
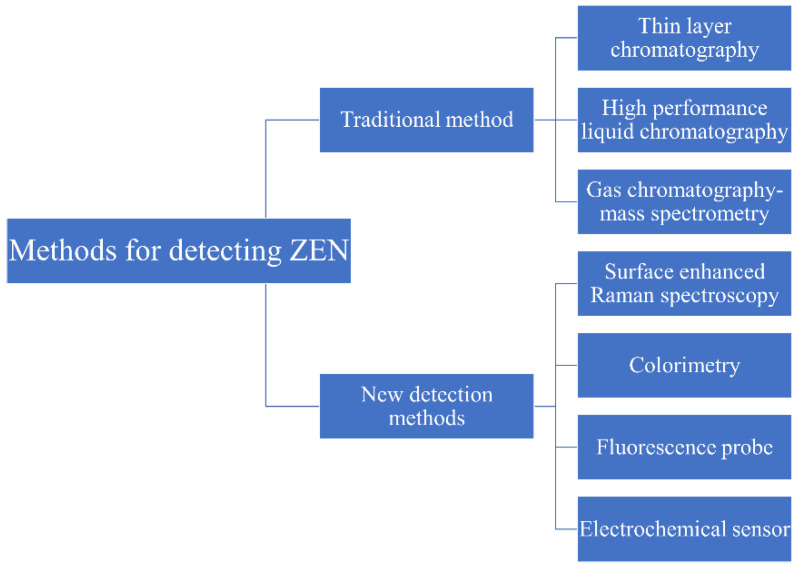
The schematic diagram of the method for realizing ZEN detection.

At present, there are many methods that can be used to detect ZEN. These methods can be roughly divided into two categories: traditional detection methods and new detection methods (Figure 2) [45,46].

### 2.1. Overview of Traditional Detection Methods of ZEN

In the past few decades, some traditional methods, such as thin-layer chromatography, high-performance liquid chromatography, and gas chromatography-tandem mass spectrometry, have been widely used to achieve quantitative detection of ZEN [47,48,49,50]. Although they have a high sensitivity and accuracy, they usually require expensive instruments, professional operators, and cumbersome pretreatment steps [51,52,53].

The detection of ZEN by thin-layer chromatography (TLC) is a common separation and analysis technology. The principle is based on the solubility difference of ZEN in different solvent systems, and the samples are separated on the thin-layer chromatography plate. After the sample was expanded on a thin-layer plate coated with an appropriate stationary phase, ZEN and other components were separated according to their physical and chemical properties, and qualitative or quantitative analysis was performed by comparing the retention factors (Rf values) of the standard substances [54]. Hadiani et al. [55] analyzed ZEN in corn samples by TLC. ZEN was extracted by chloroform, purified by silica gel column, separated by TLC plate, and quantified by density measurement. The recovery rate was 92%. The detection limit of ZEN on the TLC plate was 100 ng/g. TLC has the advantages of simple operation, low cost, and high detection sensitivity, which is especially suitable for preliminary screening and rapid detection of large quantities of samples. However, the resolution and quantitative accuracy of this method are relatively low, and it needs to be combined with other analytical methods (such as high-performance liquid chromatography) to improve accuracy.

High-performance liquid chromatography (HPLC) detection of ZEN is a common and efficient analytical technique [56,57]. The principle is to separate ZEN from other substances in the sample by liquid chromatography column [58]. In the process of liquid chromatography detection, the sample is pushed through the liquid mobile phase, and different components are separated by the stationary phase. ZEN has different retention times in the chromatographic column according to its chemical properties and different interaction forces with other components. The effluent was monitored by a detector (such as an ultraviolet detector), and ZEN could be quantitatively analyzed in combination with the standard curve. Bozkurt et al. [59] used high-performance liquid chromatography-fluorescence detector to determine ZEN, using trifluoromethanesulfonyl and trifluoromethanesulfonyl as extractants. Under the optimal conditions, the linear range of ZEN was 1–750 μg/L, and the detection limit was 0.25 μg/L. The method was successfully applied to beer and grain samples. Liquid chromatography has the advantages of high resolution, high sensitivity, and accurate quantification, which is especially suitable for the detection of complex matrix samples. However, liquid chromatography requires expensive equipment and operational technical requirements, and the detection time is relatively long.

The detection of ZEN by gas chromatography-tandem mass spectrometry (GC-MS/MS) is a highly sensitive analytical technique that combines the separation ability of gas chromatography and the identification ability of mass spectrometry [60,61,62,63,64]. Firstly, the sample was separated in a gas chromatography column, and ZEN was separated from other components according to their volatile differences. Subsequently, the separated ZEN was ionized in the mass spectrometer to generate characteristic ions, and the target substance was quantified by tandem mass spectrometry through multi-stage mass spectrometry analysis. Luo et al. [65] established a GC-MS method for the detection of ZEN and its five derivatives in feed. In this method, the samples were purified by immunoaffinity column, and the target compounds were analyzed by GC-MS after silanization and derivatization of the eluent, and the matrix effect was corrected by isotope internal standard. The six analytes showed good linear relationships in the range of 2–500 ng/mL. The limits of detection (LOD) and limits of quantitation (LOQ) were less than 1.5 μg/kg and 5.0 μg/kg, respectively. However, although GC-MS/MS is suitable for the detection of low concentrations of ZEN, it requires a more complicated sample pretreatment process, the equipment cost is high, and the operation technical requirements are strict.

### 2.2. Overview of New Detection Methods of ZEN

The new detection methods of ZEN mainly include surface-enhanced Raman spectroscopy, colorimetry, fluorescence probe method, and electrochemical sensors [66,67,68,69].

**Figure 3:**
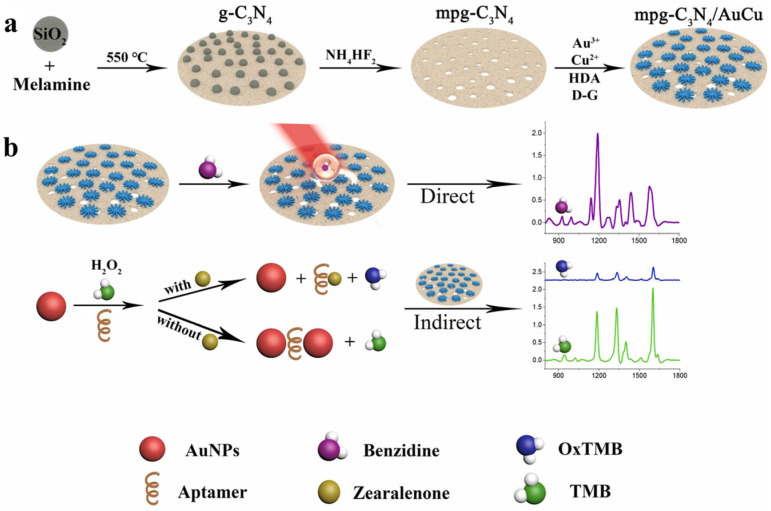
Schematic illustration of the preparation principle of mpg-C_3_N_4_/AuCu membrane for analysis of (**a**) benzidine and (**b**) zearalenone [74].

Surface-enhanced Raman spectroscopy (SERS) detection of ZEN is a highly sensitive spectral analysis technology. Its principle is based on the Raman scattering phenomenon, that is, the frequency change occurs after the interaction between the molecule and the incident light [70,71,72,73]. By forming a surface enhancement effect on the surface of metal nanomaterials, SERS can significantly improve the Raman signal so that low concentrations of ZEN can also be effectively detected. For example, Ge et al. [74] prepared mesoporous graphite phase carbon nitride (mpg-C_3_N_4_/AuCu) hybrid films doped with metal nanoalloys for SERS analysis of benzidine and zearalenone in food (Figure 3). The material combines the localized surface plasmon resonance effect of AuCu nanoalloys and the semiconductor properties of mpg-C_3_N_4_, which can be used for direct determination of benzidine and indirect determination of ZEN by 3,3′,5,5′-tetramethylbenzidine. The detection limits of this method for benzidine and zearalenone were 0.14 μg/L and 0.03 μg/L, respectively, which provided a new idea for the design of high-performance SERS substrates. SERS technology has the advantages of high sensitivity, rapid analysis, and no complex sample pretreatment, which is very suitable for on-site rapid detection. The advantages of SERS technology include high sensitivity, rapid analysis, and simple sample processing, which enable it to detect target substances at low concentrations and make it suitable for rapid on-site detection. However, it also has some disadvantages, such as strong dependence on metal nanomaterials, high requirements for material preparation, and matrix effects in complex samples that may interfere with the analysis results. Therefore, these problems need to be optimized in practical applications.

**Figure 4:**
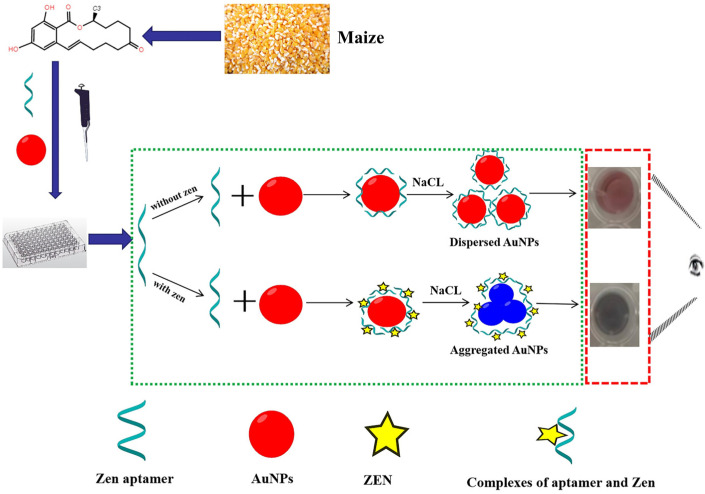
Scheme of colorimetric aptamer sensor for ZEN detection. In the absence of ZEN, the color of the designed aptamer sensor is red. In the presence of ZEN, the color of the aptamer sensor is blue [80].

The colorimetric method is a method for quantifying the concentration of a substance by measuring the color change of the sample after reacting with a specific reagent. In the detection of ZEN, common colorimetric methods include lateral flow analysis (LFA) and enzyme-linked immunosorbent assay (ELISA) [75,76,77,78,79]. LFA can realize qualitative analysis by visual color lines or quantitative analysis by signal strength, which is an effective means of rapid detection. In ELISA, the enzyme-labeled antibody is combined with ZEN to produce a color reaction, which is quantitatively analyzed by the change of absorbance. For example, Zhang et al. [80] proposed a smartphone-assisted aptamer sensor based on a colorimetric method for rapid, low-cost, and sensitive detection of ZEN in corn (Figure 4). The sensor uses ZEN aptamer as the recognition element and gold nanoparticles (AuNPs) as the indicator, combined with colorimetry and smartphones for detection. Figure 2 shows the working process of the sensor. The sensor has a good linear response in the concentration range of 5–300 ng/mL, the detection limit is 5 ng/mL, and it can successfully measure ZEN in corn samples. In addition, the entire detection process takes only 15 min. Compared with traditional methods, it has the advantages of simplicity, rapidity, and low cost and is especially suitable for the detection of ZEN in cereals. However, it should be noted that interfering substances may have a greater impact on the results of colorimetry. Therefore, sample pretreatment and reaction conditions should be optimized during the operation to ensure the accuracy of the test results. However, due to the great influence of interfering substances on the colorimetric method, attention should be paid to the optimization of sample pretreatment and reaction conditions during the operation to ensure the accuracy of the test results.

**Figure 5:**
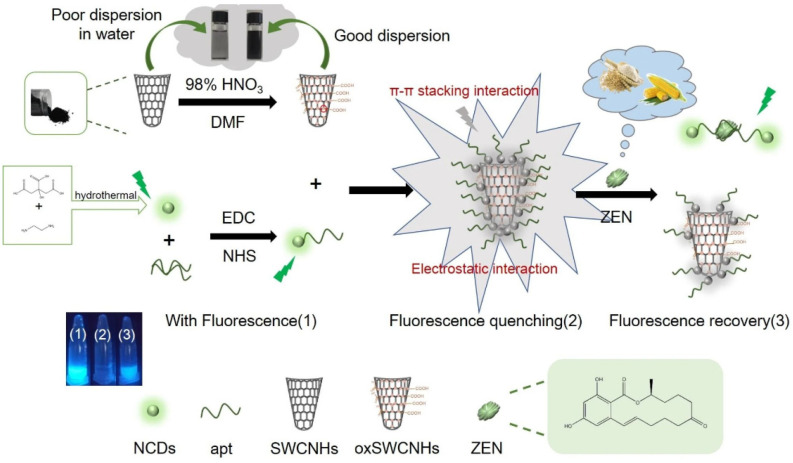
Schematic diagram of fluorescence sensor for ZEN detection [86].

The fluorescence probe method for the detection of ZEN is an analytical technique based on the interaction between specific fluorescent probes and ZEN molecules [81,82]. The principle of this method is that the fluorescence signal changes after the synthesized fluorescent probe is combined with the ZEN molecule. This change can be measured by a fluorescence spectrometer or other detection equipment to achieve quantitative analysis of ZEN [83,84,85]. Fluorescent probes are generally designed to have specific binding ability to ZEN by chemical or physical means. When ZEN molecules exist, the fluorescence emission intensity, wavelength, or fluorescence lifetime of the probe changes, and these changes can be used to accurately identify the presence and concentration of ZEN. For example, Na et al. [86] developed a rapid fluorescence sensor for the detection of mycotoxin ZEN in corn and flour by combining the fluorescence characteristics of n-doped carbon dot aptamers (NCDs-apt) and the quenching ability of oxidized single-walled carbon nanohorns (oxSWCNHs) (Figure 5). In this study, NCDs were synthesized by a one-step hydrothermal method and connected with ZEN-aptamer (ZEN-apt), which combined with oxSWCNHs to quench the fluorescence of NCDs-apt, thus forming a sensor based on the fluorescence ‘on-off’ principle. Under the optimized conditions, the sensor has a detection limit of 18 ng/mL and a linear range of 20–100 ng/mL. Through the study of possible interfering substances, the results show that the sensor has good selectivity. The recovery rate was between 99.5% and 114.3%, and the relative standard deviation (RSD) was not more than 6.5%, which showed high accuracy and precision. The study also shows that the oxSWCNHs/NCDs-apt fluorescent sensor has good sensitivity, selectivity, simplicity, and efficiency in the detection of ZEN and can be effectively applied to the detection of ZEN residues in actual food samples. The excellent fluorescence quenching performance and good water dispersibility of oxSWCNHs make the sensor perform well in actual food samples. In addition, oxSWCNHs can also be used as a general fluorescence quencher for other fluorescent dyes. Therefore, this study provides a practical fluorescence sensor method for the detection of toxins in food, which has broad application potential. However, the stability of fluorescent probes is a key issue. In complex food matrices or environmental samples, fluorescent probes may be interfered with or degraded, resulting in inaccurate detection results. Secondly, the selectivity of some fluorescent probes to ZEN may not be strong enough, and it is easy to bind non-specifically with other molecules, which affects the reliability of detection. In addition, the photobleaching effect of fluorescent probes is also a major problem. Long-term use may lead to the attenuation of fluorescence intensity, thereby reducing the detection sensitivity.

Electrochemical sensors achieve quantitative analysis of ZEN by monitoring changes in electrochemical signals such as current, voltage, or resistance [87,88,89,90]. The principle is based on the interaction between ZEN and electrode surface modification materials. Usually, antibodies, aptamers, or nanoenzymes are used as recognition elements [91,92]. When ZEN binds to these recognition materials, the resulting electrochemical signal changes can be measured by an electrochemical workstation [93,94]. Electrochemical sensors have the characteristics of high sensitivity, fast response, and simple operation, which are suitable for on-site real-time detection, and the equipment cost is low [95,96,97]. Its advantages also include strong selectivity, reproducibility, and low sample demand. Although electrochemical sensors have shown great potential in many industrial fields, the performance of electrochemical sensors for detecting ZEN still faces many challenges in laboratory research due to the influence of electrode materials, reaction conditions, and sensor stability.

## 3. Research Progress of Electrochemical Sensors in ZEN Detection

In the process of ZEN detection, according to the different recognition elements of electrochemical sensors, they can be mainly divided into aptamer electrochemical sensors, immune-electrochemical sensors, and nanoenzyme electrochemical sensors. These sensors have their own unique recognition mechanisms and advantages.

### 3.1. Aptamer Electrochemical Sensor

By introducing the aptamer into the sensor substrate, the electrochemical aptamer sensor can trigger electron transfer through the specific interaction between the aptamer and the target, thereby generating an electrochemical signal response [98]. These sensors provide a simple, rapid, portable, disposable and economical detection platform for mycotoxins by using electrochemical impedance spectroscopy (EIS), cyclic voltammetry (CV), differential pulse voltammetry (DPV), and square wave voltammetry (SWV). According to the different detection principles, aptamer sensors can be divided into non-competitive aptamer sensors and competitive aptamer sensors.

**Figure 6:**
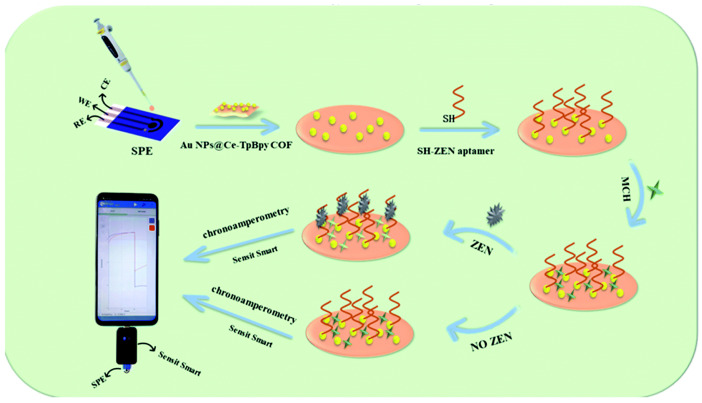
Preparation process of ZEN aptasensor [99].

Without the introduction of haptens, electrochemical aptamer sensors based on non-competitive forms have received extensive attention because of their ability to directly output potential, current, and impedance signals, showing the advantages of direct detection. When ZEN binds to the aptamer immobilized on the electrode, the conformation of the aptamer changes, which directly affects the electron transfer performance of the sensing platform. Chen et al. [99] proposed an aptamer sensor (Figure 6) combining a portable u-disk electrochemical workstation and a screen-printed electrode for quantitative detection of ZEN. In the process of electrode preparation, 1,3,5-triformylphloroglucinol (Tp) and 2,2′-bipyridine-5,5′-diamine (Bpy) were first reacted in a mixed solvent of dimethylacetamide, o-dichlorobenzene, and water acetic acid to synthesize TpBpy COF, which was heated at 120 °C for 3 days to obtain a dark red solid. Then, cerium nitrate hexahydrate was added to react with methanol to obtain a Ce-TpBpy covalent organic framework (COF). Subsequently, HAuCl4 was reduced to nano-gold sol using the gold nanoparticle sol method and combined with Ce-TpBpy COF to form Au NPs@Ce-TpBpy COF composites. Finally, the composite material was mixed with chitosan to prepare an electrode substrate, and a zearalenone aptamer sensor was constructed by aptamer modification. The sensor works by chronoamperometry. When there is no ZEN in the system, the catalytic current of Ce-TpBpy COF to hydrogen peroxide is higher. However, when ZEN is introduced into the system, ZEN specifically binds to the aptamer and inhibits electron transfer, thereby reducing the current of AuNPs@Ce-TpBpy COF catalytic reduction of H_2_O_2_. By measuring this current change, quantitative detection of ZEN can be achieved. The sensor exhibits good sensitivity, selectivity, and repeatability, with a linear range of 1 pg/mL–10 ng/mL and a detection limit of 0.389 pg/mL. It provides a new strategy for rapid, low-cost, and real-time detection of ZEN.

**Figure 7:**
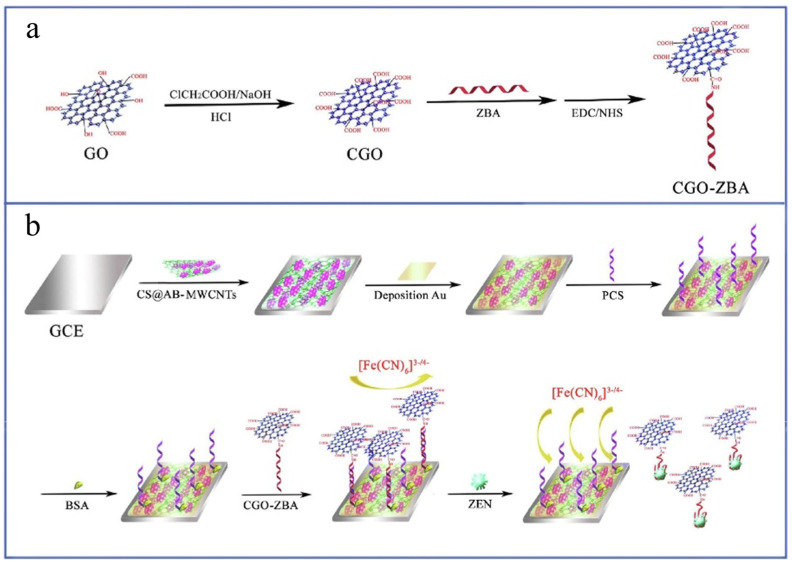
(**a**) Preparation of the CGO-ZBA bioconjugate; (**b**) Schematic diagram for the fabrication of ZEN aptasensor [100].

The electrochemical aptamer sensor based on the non-competitive type is an indirect detection strategy, which is especially suitable for small molecule compounds such as ZEN. During the detection process, mycotoxins compete with their aptamers through high-affinity binding, and this reaction is the key to the detection. By monitoring the change of electrochemical signal, the content of ZEN can be quantified. Generally, indirect competitive detection methods are combined with signal amplification techniques, such as enzymatic and nanomaterial methods, to improve the sensitivity to trace mycotoxins. Based on the target-induced amplification strategy, Mu et al. [100] constructed a highly sensitive ZEN signal aptamer sensor (Figure 7). Chitosan acetylene black multi-walled carbon nanotubes (CS@AB-MWCNTs) nanocomposites with large specific surface area and excellent conductivity were synthesized as sensing platforms. First, when synthesizing CS@AB-MWCNTs nanocomposites, CS was dissolved in HAc solution, added with AB powder, and dispersed by ultrasonication; then mixed with MWCNTs and stirred; and finally centrifuged, washed, and stored. Then, the CGO-ZBA bioconjugate was prepared by ultrasonic treatment of graphene oxide, adding NaOH and chloroacetic acid, then cross-linking with NHS and EDC, and finally adding ZBA solution to form the conjugate. The fabrication process of the sensor includes polishing and electrolytic cleaning of the bare electrode GCE to ensure that the electrode surface is clean and preparing CS@AB-MWCNTs suspension to coat on the electrode surface. This process includes electrochemical deposition, PCS solution incubation, and BSA blocking steps to complete the electrode modification and incubation of bioconjugates. In terms of detection principle, after ZEN incubation, the signal response of the current increased sharply because the induction of ZEN caused CGO-ZBA to fall off from the electrode surface, and the higher the concentration of ZEN, the greater the increase in the response signal. This increase in signal response is closely related to the electrochemical signal change of [Fe(CN)_6_]^3−/4−^. The detection range of the sensor for ZEN was 10 fg/mL–1 ng/mL, and the detection limit was 3.64 fg/mL. The method has high sensitivity, good selectivity, stability, and reproducibility.

### 3.2. Immuno-Electrochemical Sensor

An antibody-based immune-electrochemical sensor is a sensor that uses the principle of highly specific binding between an antibody and specific target molecules to achieve quantitative detection of target substances. In terms of working principle, antibody-based electrochemical sensors usually consist of three parts: electrodes, antibody molecules, and target molecules to be detected. Firstly, specific antibody molecules are immobilized on the electrode surface by covalent bonds or physical adsorption. Then, when the sample containing the target molecule contacts the sensor, the target molecule binds to the antibody to form an antibody-antigen complex. This binding process changes the charge distribution on the electrode surface, causing changes in electrochemical signals such as current, impedance, or potential. By monitoring these signal changes, the concentration of the target molecule can be quantitatively analyzed. Due to the high specificity and affinity of antibodies, antibody-based electrochemical sensors can accurately identify and detect trace amounts of target substances in complex samples with high selectivity and sensitivity.

**Figure 8:**
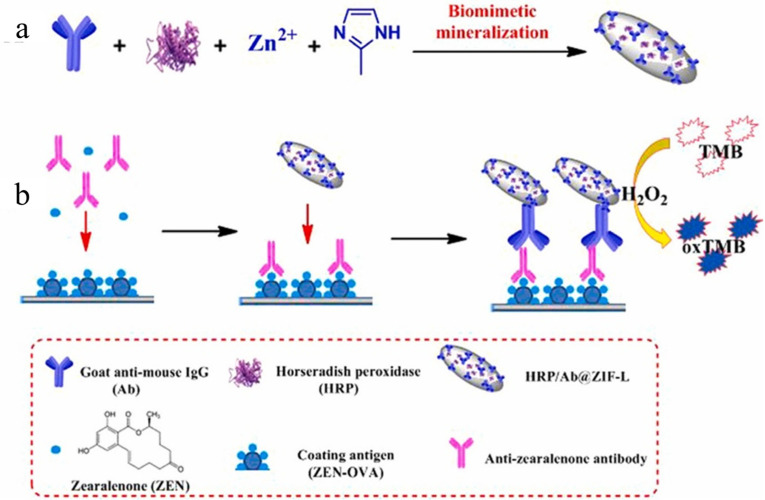
(**a**) Schematic representation of the synthetic process for HRP/Ab@ZIF-L and (**b**) the corresponding HRP/Ab@ZIF-L-based ELISA for the detection of ZEN [15].

Liu et al. [15] proposed a novel immunoassay system based on metal–organic framework (Figure 8). The system uses a metal–organic framework encapsulated with horseradish peroxidase and goat anti-mouse IgG antibody (HRP/Ab@ZIF-L) as a marker for ultrasensitive detection of ZEN in agricultural products. Specifically, the preparation process of HRP/Ab@ZIF-L included first mixing 160 μL HRP solution with a concentration of 10 mg/mL, 150 μL Ab solution with a concentration of 10 mg/mL, and 1.2 mL Zn(NO_3_)_2_·6H_2_O solution with a concentration of 1.4 mol/L and stirring for 15 min. Then, the mixed solution was quickly added to 1.5 mL of 2-methylimidazole solution with a concentration of 0.2 mol/L and stirred at room temperature for 2 h. Subsequently, centrifuged at 10,000 rpm for 10 min, washed three times with deionized water, and dried overnight at 60 °C in vacuum. The synthesis method ensures the stability and catalytic performance of the HRP/Ab@ZIF-L metal-organic framework. The detection mechanism is based on the dual function of HRP/Ab@ZIF-L; it can not only recognize the specific capture ability of the corresponding antigen but also generate an amplification signal through the catalysis of horseradish peroxidase to improve the detection sensitivity. Under the optimal conditions, the detection limit of this method for ZEN was 0.5 ng/L, and the sensitivity was about 126 times that of conventional immunoassay. At the same time, there was a good linear relationship in the range of 0.5 ng/L–0.476 μg/L of ZEN concentration, and the recovery rate of ZEN in corn and wheat samples was 84.50–96.70%. This method has the potential for rapid and ultra-sensitive detection of ZEN in agricultural products.

**Figure 9:**
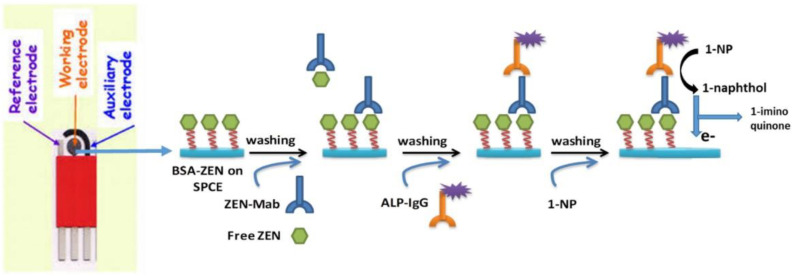
Schematic representation of the electrochemical immunosensors principle [101].

Goud et al. [101] fixed bovine serum albumin coupled with ZEN on the surface of a screen-printed carbon electrode (SPCE) and activated the electrode surface by cyclic voltammetry (Figure 9). Then, the mixture of 20 μg/mL BSA-ZEN solution with 100 mM EDC and 25 mM NHS solution was used for electrode surface immobilization, and the reaction time was 1 h. In the immunoassay step, the electrode surface was first blocked with 100 μL of 1% BSA solution for 1 h to reduce non-specific binding. The immunosensor is based on the principle of competitive immune response, in which BSA-ZEN immobilized on the electrode surface competes with free ZEN molecules to form a ZEN-MAb antibody complex. After the addition of ALP-labeled secondary antibody, the enzymatic product generated by the reaction was converted from 1-naphthol (1-NP) to 1-iminoquinone, generating an electrochemical signal on the surface of SPCE. Due to the existence of different concentrations of ZEN, the competitive effect reduces the amount of immobilized ZEN molecules bound to anti-ZEN-MAb, which in turn leads to a decrease in the number of ZEN-MAb reacting with ALP-labeled secondary antibody. Therefore, the accumulation of 1-NP conversion products on the electrode surface is reduced, resulting in a decrease in the oxidation peak current. Through the principle of this indirect immunosensor, a calibration curve between ZEN concentration and DPV oxidation peak current can be established to quantitatively detect ZEN. The detection limit of the sensor is 0.25 ng/mL, with good specificity, selectivity, reproducibility, and sensitivity. The recovery of ZEN in beer and wine was 89–97%. This immunosensing principle has broad application prospects in analyte detection technology.

### 3.3. Nanoenzyme Electrochemical Sensor

Nanozymes are nanomaterials with enzyme-like catalytic activity, which are usually composed of metals, metal oxides, carbon-based materials, etc. They can simulate the function of natural enzymes, catalyze chemical reactions, and have a high stability, are adjustable, and have a wide application potential. Compared with natural enzymes, nanozymes not only have strong environmental adaptability but also can regulate their catalytic performance by changing the structure, composition, and morphology of nanomaterials, which are widely used in biosensors, environmental governance, disease diagnosis, and other fields. The nanozyme electrochemical sensor makes full use of the enzyme-like activity of nanomaterials. By immobilizing nanozymes with enzyme-like catalytic ability on the electrode surface, it can effectively react with specific substrates to produce stable electrochemical signals. Compared with natural enzymes, these nanozymes can not only simulate their catalytic performance but also have better high-temperature resistance, acid and alkali resistance, stability, and adjustability, so that they can maintain high catalytic activity in various extreme environments. There is a linear relationship between the electrochemical signal intensity generated during this reaction and the substrate concentration, so the concentration of the substrate can be accurately measured. With this principle, the nanoenzyme electrochemical sensor has excellent high sensitivity and high selectivity, which can detect target molecules at extremely low concentrations and can accurately distinguish the required substances in complex samples. This kind of sensor is widely used in biomedicine, environmental monitoring, food safety, drug detection, and other fields, especially in real-time and rapid detection, showing great potential. In the field of food safety, it can be used to detect trace amounts of harmful substances, pesticide residues, and pathogenic bacteria in food; in terms of environmental monitoring, it can effectively detect toxic substances and pollutants in water quality and air; and in clinical diagnosis, nanoenzyme electrochemical sensors can help quickly diagnose disease markers and provide instant feedback. With the continuous advancement of technology, the performance of these sensors has gradually improved, and the application range has become wider and wider. It has a very large commercial and practical prospect, especially in the context of increasing demand for on-site monitoring and low-cost, high-efficiency detection technology.

**Figure 10:**
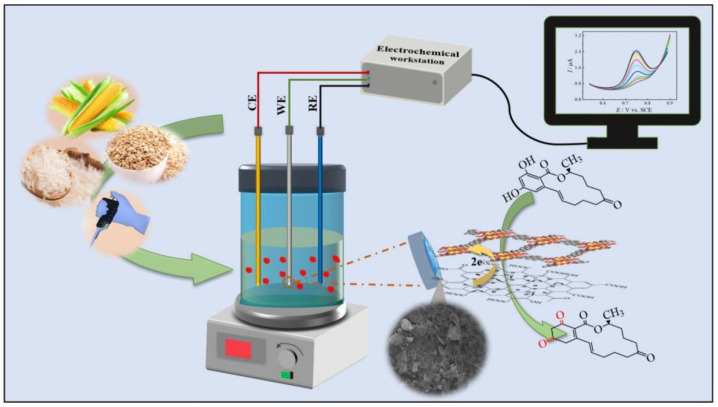
Schematic illustration of detection and electrocatalytic mechanism of ZEN using nanohybrid sensor based on Cu-MOF/Fe_3_O_4_-GO/GCE [102].

Zeng et al. [102] prepared a nanoenzyme electrochemical sensor (Figure 10) based on a copper-based metal–organic framework (Cu-MOF) and a magnetic Fe_3_O_4_-graphene oxide (Fe_3_O_4_-GO) modified electrode by layer-by-layer assembly technique for rapid determination of ZEN in breakfast and corn flour. Cu-MOF has high porosity, which can effectively improve the specific surface area and electron transfer ability of the nanoenzyme sensor. Under the optimal conditions, the sensor can accurately quantify ZEN in the linear range of 159.2–2865.2 ng/mL with a detection limit of 23.14 ng/mL.

**Figure 11:**
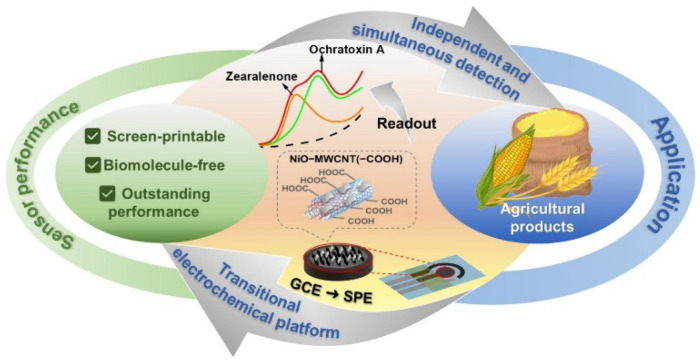
The schematic illustration of synthesis of NiO–MWCNT(–COOH) and fabrication of NiO–MWCNT(–COOH)-based electrode [103].

Veenuttranon et al. [103] developed a biomolecule-free nanoenzyme electrochemical sensor (Figure 11), which can simultaneously detect ZEN and OTA based on the synergistic effect of nickel oxide (NiO) and carboxylated carbon nanotubes (MWCNT(–COOH)). The sensor realized the potential-resolved selective detection of ZEN and OTA for the first time, with excellent sensitivity, selectivity, reusability, and stability. The sensor fabricated by screen printing technology shows its commercial potential and has been successfully applied to the detection of mycotoxins in corn flour and wheat flour, indicating its good prospect in the monitoring of agricultural product pollution.

**Figure 12:**
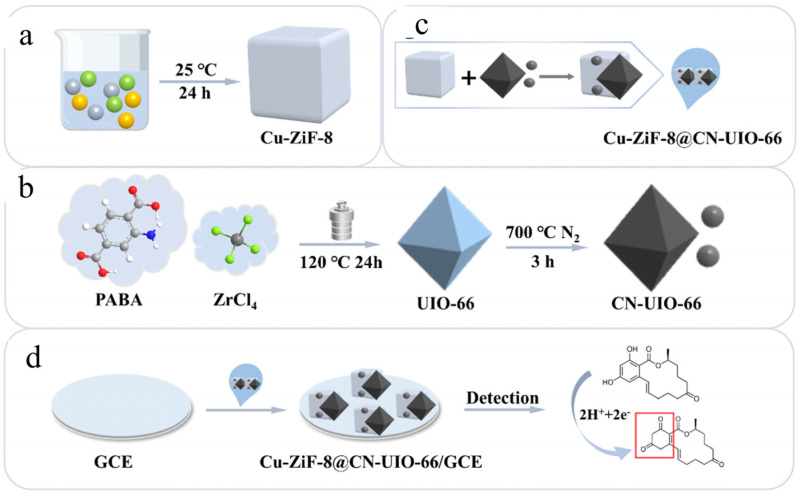
Preparation process diagram of Cu-ZiF-8@CN-UIO-66/GCE [104].

Lin et al. [104] developed a nanozyme electrochemical sensor based on Cu-doped ZIF-8 and calcined UIO-66 for rapid and sensitive detection of ZEN mycotoxins in vegetable oil (Figure 12). The fabrication process of the sensor includes the synthesis of Cu-doped Zeolitic imidazolate framework-8 (Cu-ZIF-8) and calcined Universitetet i Oslo-66 (CN-UIO-66), and then the nanozyme electrochemical sensor was prepared by ultrasonic synthesis. The introduction of copper improves the catalytic activity of ZIF-8 by exerting electronic influence. At the same time, the synergistic effect of calcined UIO-66 and Cu-ZIF-8 further enhances the performance of the sensor, especially improving the electrical response and conductivity, thus significantly improving the recognition ability of ZEN. The prepared nanozyme electrochemical sensor can specifically electrooxidize ZEN, which is an irreversible electrochemical process involving two electrons and two protons. In this process, the resorcinol ring is likely to undergo an oxidation reaction, in which the hydroxyl group of resorcinol is irreversibly oxidized to a benzoquinone structure. Through the synergistic effect of copper introduction and calcined UIO-66, the catalytic efficiency and electron transfer performance of the sensor have been significantly improved, thus showing higher sensitivity and faster response in the electrochemical detection process. The linear range of the sensor was 10 ng/mL–25 μg/mL, and the detection limit was 0.6 ng/mL. Compared with the traditional method, the sensor not only shortens the detection time to only 10 min but also has a lower detection limit and good anti-interference ability. In the actual sample detection, the sensor is highly consistent with the HPLC detection results, and the recovery rate is between 95.9% and 102.2%, showing its wide application prospect.

**Figure 13:**
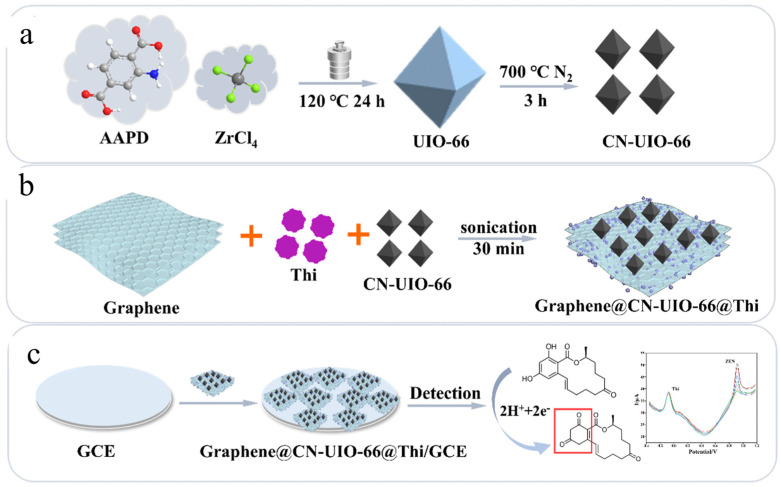
Preparation process diagram of Graphene@CN-UIO-66@Thi/GCE [105].

Lin et al. [105] proposed a novel nanoenzyme ratiometric electrochemical sensor based on graphene, calcined UIO-66, and thionine for rapid and sensitive detection of ZEN in vegetable oil and its derivatives (Figure 13). Through the conductivity and specific surface area of graphene, combined with the stable structure and catalytic activity of calcined UIO-66 and the signal amplification effect of thionine, the sensor can achieve high-sensitivity detection of ZEN in the linear range from 1 ng/mL to 25 μg/mL, and the minimum detection limit is 0.058 ng/mL. The sensor has good anti-interference performance for common interferences in vegetable oils, such as vitamin E and stigmasterol, and has a high recovery rate in actual samples. The detection results are highly consistent with HPLC. This study provides an effective tool for real-time and accurate detection of ZEN in vegetable oil and its derivatives and has broad application prospects.

**Figure 14:**
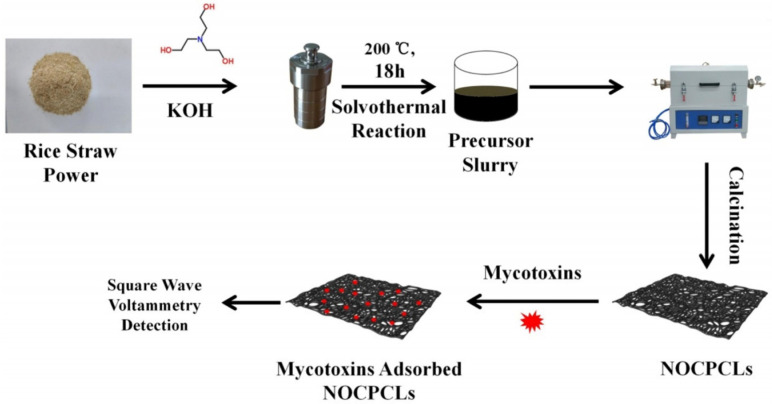
Synthetic process of N/O co-doped porous carbon lamellas (NOCPCLs) and the detection of mycotoxins [106].

Yang et al. [106] proposed a novel electrochemical sensor based on N/O co-doped porous biomass carbon (NOCPCLs) electrodes for highly sensitive detection of mycotoxins (Figure 14). In this study, rice straw was used as raw material to synthesize NOCPCLs by triethanolamine/KOH solvothermal reaction, and it was applied to electrode modification to enhance the detection ability of mycotoxins. The sensor showed excellent universality and sensitivity in the detection of various mycotoxins (such as aflatoxin B1, G1, G2, M1, zearalenone, and deoxynivalenol). The detection limits were 0.5689, 0.0504, 0.0274, 0.6141, 0.0781, and 0.0512 fg mL^−1^, respectively, and the dynamic linear range was from 0.001 to 1000 pg mL^−1^. Through density functional theory (DFT) calculations, the researchers found that the adsorption of mycotoxins by NOCPCL materials led to changes in the charge distribution on the electrode surface, which further improved the sensitivity of the sensor. The experimental results also showed that the sensor based on NOCPCLs showed high accuracy in the detection of mycotoxins in actual samples, and the recovery rate was close to 100%. Therefore, this study provides a new idea for the development of rapid, efficient, sensitive, and widely applicable electrochemical sensors and has great potential in the monitoring of mycotoxins in food and feed products.

**Figure 15:**
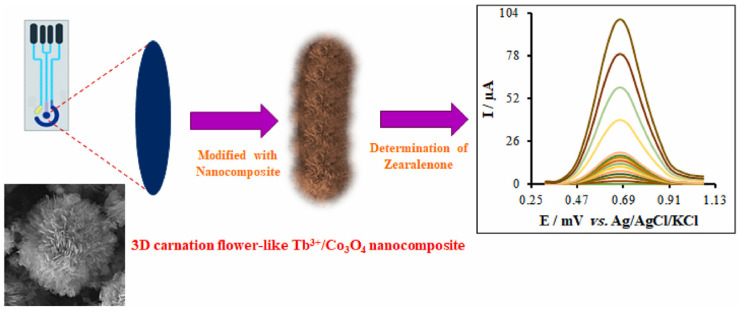
Preparation flow chart of electrochemical sensor based on a three-dimensional carnation flower-like Tb^3+^/Co_3_O_4_ nanocomposites modified with a screen-printed electrode for the detection of zearalenone [107].

Yang et al. [107] proposed a novel three-dimensional carnation-like Tb^3+^/Co_3_O_4_ nanocomposite (3D CF-L Tb^3+^/Co_3_O_4_ NC) modified screen-printed electrode (SPE) for the detection of zearalenone in liquid food samples (Figure 15). The preparation process of the sensor includes hydrothermal synthesis of 3D F-L Tb^3+^/Co_3_O_4_ NC, and its performance is evaluated by electrochemical techniques. The experimental results show that the sensor has significant high conductivity, a large surface area, and excellent stability. It can achieve the best signal response at pH 7.0, showing a wide linear dynamic range (0.001~500.0 μM) and low detection limit (0.34 nM). The ability of the sensor to successfully determine zearalenone in actual agricultural food samples was verified by electrochemical analysis such as differential pulse voltammetry, electrochemical impedance spectroscopy, and cyclic voltammetry. As a signal probe material, 3D CF-L Tb^3+^/Co_3_O_4_ NC significantly increases the load of signal molecules and achieves the effect of signal amplification, thereby enhancing the performance of the sensor. In summary, this study provides a new electrochemical sensing platform for the efficient detection of zearalenone, with good specificity, stability, and repeatability, showing the potential in practical applications.

**Figure 16:**
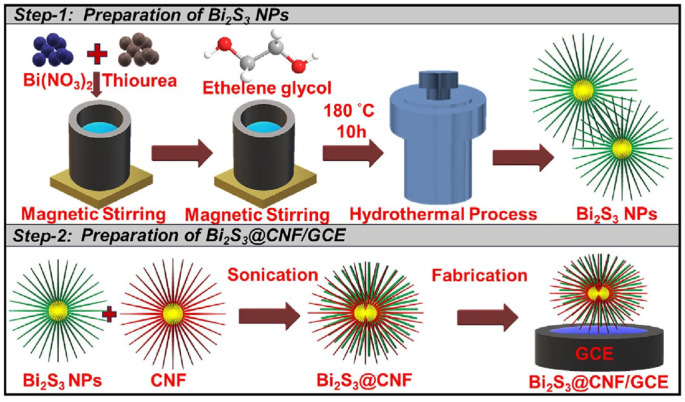
The schematic representation of the synthetic procedure of Bi2S3@CNF nanocomposite [108].

Huang et al. [108] prepared Bi_2_S_3_ nanorods by hydrothermal synthesis and combined them with carbon nanofibers (CNF) to form Bi_2_S_3_@CNF hybrid nanomaterials for the development of innovative electrochemical biosensors (Figure 16). In the specific preparation process, bismuth nitrate pentahydrate and thiourea were dissolved in distilled water, mixed with ethylene glycol, and reacted in a high-pressure sterilizer to obtain Bi_2_S_3_ precipitate. The functionalized CNF was combined with Bi_2_S_3_ in a ratio of 1:1 by ultrasonic technology to form Bi_2_S_3_@CNF nanocomposites with excellent electrochemical properties. The material showed a wide linear detection range (0.125–375.5 μM and 438–1951 μM), a low detection threshold (LOD: 0.61 μM), and high sensitivity in the detection of zearalenone. Characterization techniques, such as X-ray diffraction (XRD), field emission scanning electron microscopy (FE-SEM), and energy dispersive X-ray spectroscopy (EDX), confirmed the effective integration of Bi_2_S_3_@CNF nanocomposites in the porous CNF matrix, further indicating that the material has rich active sites and a large surface area. The successful application of this nanocomposite not only improves the detection ability of zearalenone in complex agricultural environments but also proves its practicability in the analysis of actual agricultural samples. Future research can develop more electrochemical detection devices based on this material to expand its potential in various sensor applications.

**Figure 17:**
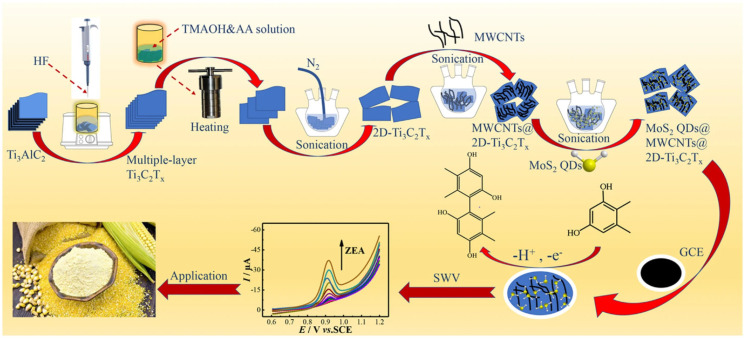
Schematic illustration of preparation and application of MoS_2_ QDs@MWCNTs@2D-Ti_3_C_2_T_x_/GCE [109].

Huang et al. [109] developed a new type of electrochemical sensor, using molybdenum disulfide quantum dots (MoS_2_ QDs) and two-dimensional titanium carbide (2D-Ti_3_C_2_T_x_ MXene) co-modified multi-walled carbon nanotubes (MWCNTs) heterostructure to sensitively detect zearalenone (ZEN) in food (Figure 17). The preparation process of the sensor includes the synthesis of multilayer Ti_3_C_2_T_x_ sheets by the etching method and the preparation of 2D-Ti_3_C_2_T_x_ sheets by the hydrothermal method. Then, MoS_2_ QDs were mixed with MWCNTs and 2D-Ti_3_C_2_T_x_, and the MoS_2_ QDs@MWCNTs@2D-Ti_3_C_2_T_x_ nanohybrids were obtained by ultrasonic treatment. Finally, the suspension droplets were added to the surface of the glassy carbon electrode by the drop casting method to obtain a modified electrode. The experimental results show that the MWCNTs modified by 2D-Ti_3_C_2_T_x_ and MoS_2_ QDs produce a synergistic signal amplification effect and have a large specific surface area and excellent conductivity, thus significantly improving the detection performance of the sensor for ZEN. Under optimized conditions, the sensor showed a wide linear range from 3.00 to 300 ng mL^−1^ and a low detection limit (LOD) of 0.32 ng mL^−1^, which was much lower than the maximum residue limit (MRL) specified by the European Union. In addition, the sensor showed good selectivity, high reproducibility (relative standard deviation of 1.1%), and excellent recovery (94.8% to 105%) in real sample analysis. This study provides an effective and valuable reference for the design of nanozyme sensors based on heterostructures.

### 3.4. Comparison of Nanozyme Sensors for ZEN Detection with Other Sensors

Table 1 lists the comparison of ZEN detection performance of different sensors. It can be seen from the table that nanozyme electrochemical sensors show significant advantages in the field of food detection. Compared with other types of sensors, nanozyme sensors have faster response time and a lower limit of detection (LOD). For example, the response time of nanozyme electrochemical sensors such as ZnO@Ag/GCE and Cu-Zif-8@CN-UIO-66/GCE is 10 min, which is much lower than other sensors, such as SERS-based lateral flow immunosensors (24 h) and soluble aptamer cross-linked hydrogel chromatographic sensors (2.75 h). In addition, the nanozyme sensor also has a low detection limit in the detection of food samples. For example, the detection limit of the Graphene@CN-UIO-66@Thi/GCE nanozyme sensor is only 0.058 ng/mL, which is lower than many other sensors, especially in complex samples (such as vegetable oil deodorizer distillate). In addition, the nanozyme sensor is flexible in design, capable of preprocessing in a relatively short time, and can perform real-time detection without complex chemical reactions or expensive equipment.

In summary, nanoenzyme electrochemical sensors have shown strong application potential in various fields, especially in food safety, environmental monitoring, and clinical diagnosis. It uses nanozymes to simulate the catalytic activity of natural enzymes, combined with advanced nanomaterial technology, which significantly improves the sensitivity, selectivity, and stability of the sensor. With the deepening of research and the continuous innovation of technology, the performance of nanoenzyme electrochemical sensors has been greatly improved, and they can still maintain excellent catalytic activity in extreme environments, providing an effective solution for on-site monitoring and real-time detection. In addition, with the increasing demand for low-cost and high-efficiency detection technologies in the market, the commercial prospects of such sensors are also growing. In the future, nanoenzyme electrochemical sensors are expected to play an important role in a wider range of applications and promote the development of various industries, especially in improving detection accuracy and response speed.

## 4. The Existing Challenges of Electrochemical Sensors Based on Nanoenzymes

Although nanoenzyme electrochemical sensors have shown broad application prospects in the fields of environmental monitoring, food safety, and biomedicine, there are still some challenges in practical applications.

### 4.1. Production Costs and Large-Scale Applications

Although nanoenzyme electrochemical sensors have great application potential, large-scale production and commercialization still face certain cost challenges. The manufacturing process of many sensors involves high-performance nanomaterials and complex preparation processes, which may lead to higher production costs. In order to realize the popularization and application of low cost and high efficiency, it is still an important goal to reduce the production cost and simplify the preparation process.

### 4.2. Control of Signal Amplification Effect

The high sensitivity of nanoenzyme sensors usually depends on signal amplification mechanisms, such as the use of nanomaterials to enhance the signal. However, the control of this amplification effect is very complex, which may lead to nonlinear response or detection error of the signal. In practical applications, how to accurately adjust the amplification effect to avoid over-amplification or unstable signals is an important challenge.

### 4.3. Safety Issues of Nanomaterials

Although the use of nanomaterials as the core component of nanoenzyme electrochemical sensors has improved performance, the biosafety and environmental impact of long-term exposure to the environment is still an important issue. Nanomaterials may have potential toxic effects on the human body or ecological environment. Therefore, ensuring the biocompatibility and environmental friendliness of nanomaterials is an important research direction.

In summary, although the nanoenzyme electrochemical sensor has great potential in practical applications, more research and technological innovation are still needed to overcome the above challenges and improve its safety and cost-effectiveness.

## 5. Conclusions and Prospects

In this paper, the application of nanoenzyme electrochemical sensors in the detection of ZEN in food was reviewed. With the increasing attention to food safety, nanoenzyme electrochemical sensors have become an important research direction in the field of zearalenone detection due to their high sensitivity, simplicity, and low cost. Traditional ZEN detection methods, such as high-performance liquid chromatography and enzyme-linked immunosorbent assay, have a high accuracy, but they are cumbersome to operate, time-consuming, and require complex experimental equipment. In contrast, the nanoenzyme electrochemical sensor shows the advantages of fast, real-time, and efficient detection, which can provide higher selectivity and sensitivity. However, although the nanoenzyme electrochemical sensor has broad application prospects in ZEN detection, it still faces some technical challenges. First of all, the high production cost of nanoenzymes and the difficulties in large-scale production limit their commercialization and widespread application. Secondly, the regulation of the signal amplification effect is still an urgent problem to be solved. How to improve the stability and sensitivity of the sensor without losing repeatability still needs to be further studied. Finally, the safety of nanomaterials also needs to be carefully considered, especially in the application of food detection. How to ensure that the sensor materials are harmless to food safety is an important direction for future research. In the future, with the continuous development of nanotechnology, material science, and bionics, nanoenzyme electrochemical sensors are expected to make breakthroughs in improving detection accuracy, reducing costs, and enhancing sensor stability. In addition, the optimization of the signal amplification effect, the improvement of nanoenzymes, and the development of new nanomaterials will provide more possibilities for the application of nanoenzyme electrochemical sensors in the detection of zearalenone. With the solution of these technical bottlenecks, the nanoenzyme electrochemical sensor is expected to become an important detection tool in food safety monitoring in the future, providing a more efficient and reliable solution for ensuring the health of consumers. In summary, the application of nanoenzyme electrochemical sensors in the detection of zearalenone has shown great potential, but further technological innovation and optimization are still needed in many aspects.

## Figures and Tables

**Table 1:** Performance comparison between different sensors for ZEN detection.

Sensor	Sensor Type	Actual Samples	Preprocessing Time	LOD (ng mL^−1^)	Reference
oxSWCNHs/NCDs-apt fluorescence sensor	fluorescence sensor	corn, flour	4 h	18 ng mL^−1^	[86]
SERS-based lateral flow immunosensor	SERS	corn	24 h	3.6 ng mL^−1^	[12]
Multi-LFIA	fluorescence	wheat, corn, and feed	2.5 h	1.74 ng mL^−1^	[110]
NiO-MWCNT(-COOH)/GCE	Nanozyme electrochemical sensor	feedstuffs and foodstuffs	30 min	6 ng mL^−1^	[103]
Aptamer-Cross-Linked hydrogel	colorimetric	corn, beer	2.75 h	0.98 ng mL^−1^	[111]
Fluorescent copper nanocluster sensor based on TdT amplification	fluorescence sensor	none	2 h	0.1 ng mL^−1^	[112]
Chemiluminescence-based aptasensor	fluorescence sensor	corn, wheat	50 min	2.85 ng mL^−1^	[113]
Disposable aptasensing chip	Non-competitive aptamer electrochemical sensor	corn starch	14 h	5.38 ng mL^−1^	[114]
ITO_#c_-CdIn_2_S_4_/V–MoS_2_-Apt3	fluorescence sensor	corn starch	2.5 h	0.033 ng mL^−1^	[115]
ZnO@Ag/GCE	Nanozyme electrochemical sensor	vegetable oil deodorizer distillate	10 min	8 ng mL^−1^	[116]
Cu-ZiF-8@CN-UIO-66/GCE	Nanozyme electrochemical sensor	vegetable oil deodorizer distillate, vegetable oil	10 min	0.6 ng mL^−1^	[104]
Graphene@CN-UIO-66@Thi/GCE	Nanozyme electrochemical sensor	vegetable oil deodorizer distillate, vegetable oil	10 min	0.058 ng mL^−1^	[105]

## Data Availability

No new data were created or analyzed in this study. Data sharing is not applicable to this article.
